# Supplementary material for: Ancestral gene synteny reconstruction improves extant species scaffolding
Source: BMC Genomics. 2015 Oct 2;16(Suppl 10):S11. doi: 10.1186/1471-2164-16-S10-S11 (PMC4603332; doi:10.1186/1471-2164-16-S10-S11)
Supplement: Additional File 1 — Figure 10 ARt-DeCo dynamic programming scheme. Recurrence formulas used in ARt-DeCo to reconstruct adjacencies in ancestral and extant genomes. [file 1471-2164-16-S10-S11-S1.pdf]

1. $E(v_1) = \mathsf{Extant}$ and $E(v_2) = \mathsf{Extant}$:
$$c_1(v_1, v_2) = -\log_b(P(v_1 \sim v_2))$$
$$c_0(v_1, v_2) = -\log_b(1 - P(v_1 \sim v_2))$$
2. $E(v_1) = \mathsf{GLoss}$ and $E(v_2) \in \{\mathsf{Extant}, \mathsf{Spec}, \mathsf{GDup}\}$:
$$c_1(v_1, v_2) = c_0(v_1, v_2) = 0$$
3. $E(v_1) \in \{\mathsf{Extant}, \mathsf{Spec}, \mathsf{GDup}\}$ and $E(v_2) = \mathsf{GLoss}$:
$$c_1(v_1, v_2) = c_0(v_1, v_2) = 0$$
4. $E(v_1) = \mathsf{GLoss}$ and $E(v_2) = \mathsf{GLoss}$:
$$c_1(v_1, v_2) = c_0(v_1, v_2) = 0$$
5. $E(v_1) \in \{\mathsf{Extant}, \mathsf{Spec}\}$ and $E(v_2) = \mathsf{GDup}$:
$$c_1(v_1, v_2) = \min \begin{cases} c_1(v_1, b_{v_2}) + c_0(v_1, a_{v_2}), & c_0(v_1, b_{v_2}) + c_1(v_1, a_{v_2}), \\ c_1(v_1, b_{v_2}) + c_1(v_1, a_{v_2}) + \mathsf{Gain}, & c_0(v_1, b_{v_2}) + c_0(v_1, a_{v_2}) + \mathsf{Br} \end{cases}$$
$$c_0(v_1, v_2) = \min \begin{cases} c_0(v_1, b_{v_2}) + c_0(v_1, a_{v_2}), & c_0(v_1, b_{v_2}) + c_1(v_1, a_{v_2}) + \mathsf{Gain}, \\ c_1(v_1, b_{v_2}) + c_0(v_1, a_{v_2}) + \mathsf{Gain}, & c_1(v_1, b_{v_2}) + c_1(v_1, a_{v_2}) + 2\mathsf{Gain} \end{cases}$$
6. $E(v_1) = \mathsf{GDup}$ and $E(v_2) \in \{\mathsf{Extant}, \mathsf{Spec}\}$:
$$c_1(v_1, v_2) = \min \begin{cases} c_1(a_{v_1}, v_2) + c_0(b_{v_1}, v_2), & c_0(a_{v_1}, v_2) + c_1(b_{v_1}, v_2), \\ c_1(a_{v_1}, v_2) + c_1(b_{v_1}, v_2) + \mathsf{Gain}, & c_0(a_{v_1}, v_2) + c_0(b_{v_1}, v_2) + \mathsf{Br} \end{cases}$$
$$c_0(v_1, v_2) = \min \begin{cases} c_0(a_{v_1}, v_2) + c_0(b_{v_1}, v_2), & c_0(a_{v_1}, v_2) + c_1(b_{v_1}, v_2) + \mathsf{Gain}, \\ c_1(a_{v_1}, v_2) + c_0(b_{v_1}, v_2) + \mathsf{Gain}, & c_1(a_{v_1}, v_2) + c_1(b_{v_1}, v_2) + 2\mathsf{Gain}, \end{cases}$$
7. $E(v_1) = \mathsf{Spec}$ and $E(v_2) = \mathsf{Spec}$:
$$c_1(v_1, v_2) = \min \begin{cases} c_1(a_{v_1}, b_{v_2}) + c_1(b_{v_1}, a_{v_2}), & c_1(a_{v_1}, b_{v_2}) + c_0(b_{v_1}, a_{v_2}) + \mathsf{Br}, \\ c_0(a_{v_1}, b_{v_2}) + c_1(b_{v_1}, a_{v_2}) + \mathsf{Br}, & c_0(a_{v_1}, b_{v_2}) + c_0(b_{v_1}, a_{v_2}) + 2\mathsf{Br}, \\ c_1(a_{v_1}, a_{v_2}) + c_1(b_{v_1}, b_{v_2}), & c_1(a_{v_1}, a_{v_2}) + c_0(b_{v_1}, b_{v_2}) + \mathsf{Br}, \\ c_0(a_{v_1}, a_{v_2}) + c_1(b_{v_1}, b_{v_2}) + \mathsf{Br}, & c_0(a_{v_1}, a_{v_2}) + c_0(b_{v_1}, b_{v_2}) + 2\mathsf{Br} \end{cases}$$
$$c_0(v_1, v_2) = \min \begin{cases} c_0(a_{v_1}, b_{v_2}) + c_0(b_{v_1}, a_{v_2}), & c_1(a_{v_1}, b_{v_2}) + c_0(b_{v_1}, a_{v_2}) + \mathsf{Gain}, \\ c_0(a_{v_1}, b_{v_2}) + c_1(b_{v_1}, a_{v_2}) + \mathsf{Gain}, & c_1(a_{v_1}, b_{v_2}) + c_1(b_{v_1}, a_{v_2}) + 2\mathsf{Gain}, \\ c_0(a_{v_1}, a_{v_2}) + c_0(b_{v_1}, b_{v_2}), & c_1(a_{v_1}, a_{v_2}) + c_0(b_{v_1}, b_{v_2}) + \mathsf{Gain}, \\ c_0(a_{v_1}, a_{v_2}) + c_1(b_{v_1}, b_{v_2}) + \mathsf{Gain}, & c_1(a_{v_1}, a_{v_2}) + c_1(b_{v_1}, b_{v_2}) + 2\mathsf{Gain}, \end{cases}$$
8. $E(v_1) = \mathsf{GDup}$ and $E(v_2) = \mathsf{GDup}$:
$$c_1(v_1, v_2) = \min \begin{cases} c_1(a_{v_1}, v_2) + c_0(b_{v_1}, v_2), \quad c_0(a_{v_1}, v_2) + c_1(b_{v_1}, v_2), \\ c_1(a_{v_1}, v_2) + c_1(b_{v_1}, v_2) + \mathsf{Gain}, \quad c_0(a_{v_1}, v_2) + c_0(b_{v_1}, v_2) + \mathsf{Br}, \\ c_1(v_1, a_{v_2}) + c_0(v_1, b_{v_2}), \quad c_0(v_1, a_{v_2}) + c_1(v_1, b_{v_2}), \\ c_1(v_1, a_{v_2}) + c_1(v_1, b_{v_2}) + \mathsf{Gain}, \quad c_0(v_1, a_{v_2}) + c_0(v_1, b_{v_2}) + \mathsf{Br}, \\ c_1(a_{v_1}, a_{v_2}) + c_1(b_{v_1}, b_{v_2}) + c_0(a_{v_1}, b_{v_2}) + c_0(b_{v_1}, a_{v_2}), \\ c_1(a_{v_1}, a_{v_2}) + c_1(b_{v_1}, b_{v_2}) + c_0(a_{v_1}, b_{v_2}) + c_1(b_{v_1}, a_{v_2}) + \mathsf{Gain}, \\ c_1(a_{v_1}, a_{v_2}) + c_1(b_{v_1}, b_{v_2}) + c_1(a_{v_1}, b_{v_2}) + c_0(b_{v_1}, a_{v_2}) + \mathsf{Gain}, \\ c_1(a_{v_1}, a_{v_2}) + c_1(b_{v_1}, b_{v_2}) + c_1(a_{v_1}, b_{v_2}) + c_1(b_{v_1}, a_{v_2}) + 2\mathsf{Gain}, \\ c_1(a_{v_1}, a_{v_2}) + c_0(b_{v_1}, b_{v_2}) + c_0(a_{v_1}, b_{v_2}) + c_0(b_{v_1}, a_{v_2}) + \mathsf{Br}, \\ c_1(a_{v_1}, a_{v_2}) + c_0(b_{v_1}, b_{v_2}) + c_0(a_{v_1}, b_{v_2}) + c_1(b_{v_1}, a_{v_2}) + \mathsf{Gain} + \mathsf{Br}, \\ c_1(a_{v_1}, a_{v_2}) + c_0(b_{v_1}, b_{v_2}) + c_1(a_{v_1}, b_{v_2}) + c_0(b_{v_1}, a_{v_2}) + \mathsf{Gain} + \mathsf{Br}, \\ c_0(a_{v_1}, a_{v_2}) + c_1(b_{v_1}, b_{v_2}) + c_0(a_{v_1}, b_{v_2}) + c_0(b_{v_1}, a_{v_2}) + \mathsf{Br}, \\ c_0(a_{v_1}, a_{v_2}) + c_1(b_{v_1}, b_{v_2}) + c_0(a_{v_1}, b_{v_2}) + c_1(b_{v_1}, a_{v_2}) + \mathsf{Gain} + \mathsf{Br}, \\ c_0(a_{v_1}, a_{v_2}) + c_1(b_{v_1}, b_{v_2}) + c_1(a_{v_1}, b_{v_2}) + c_0(b_{v_1}, a_{v_2}) + \mathsf{Gain} + \mathsf{Br}, \\ c_0(a_{v_1}, a_{v_2}) + c_0(b_{v_1}, b_{v_2}) + c_1(a_{v_1}, b_{v_2}) + c_1(b_{v_1}, a_{v_2}), \\ c_0(a_{v_1}, a_{v_2}) + c_1(b_{v_1}, b_{v_2}) + c_1(a_{v_1}, b_{v_2}) + c_1(b_{v_1}, a_{v_2}) + \mathsf{Gain}, \\ c_1(a_{v_1}, a_{v_2}) + c_0(b_{v_1}, b_{v_2}) + c_1(a_{v_1}, b_{v_2}) + c_1(b_{v_1}, a_{v_2}) + \mathsf{Gain}, \\ c_0(a_{v_1}, a_{v_2}) + c_0(b_{v_1}, b_{v_2}) + c_1(a_{v_1}, b_{v_2}) + c_0(b_{v_1}, a_{v_2}) + \mathsf{Br}, \\ c_0(a_{v_1}, a_{v_2}) + c_0(b_{v_1}, b_{v_2}) + c_0(a_{v_1}, b_{v_2}) + c_1(b_{v_1}, a_{v_2}) + \mathsf{Br}, \\ c_0(a_{v_1}, a_{v_2}) + c_0(b_{v_1}, b_{v_2}) + c_0(a_{v_1}, b_{v_2}) + c_0(b_{v_1}, a_{v_2}) + 2\mathsf{Br}, \end{cases}$$
$$c_0(v_1, v_2) = \min \begin{cases} c_0(a_{v_1}, v_2) + c_0(b_{v_1}, v_2), & c_0(a_{v_1}, v_2) + c_1(b_{v_1}, v_2) + \mathsf{Gain}, \\ c_1(a_{v_1}, v_2) + c_0(b_{v_1}, v_2) + \mathsf{Gain}, & c_1(a_{v_1}, v_2) + c_1(b_{v_1}, v_2) + 2\mathsf{Gain}, \\ c_0(v_1, a_{v_2}) + c_0(v_1, b_{v_2}), & c_0(v_1, a_{v_2}) + c_1(v_1, b_{v_2}) + \mathsf{Gain}, \\ c_1(v_1, a_{v_2}) + c_0(v_1, b_{v_2}) + \mathsf{Gain}, & c_1(v_1, a_{v_2}) + c_1(v_1, b_{v_2}) + 2\mathsf{Gain}. \end{cases}$$
