# Supplementary figures and images for: Ancestral gene synteny reconstruction improves extant species scaffolding
Source: BMC Genomics. 2015 Oct 2;16(Suppl 10):S11. doi: 10.1186/1471-2164-16-S10-S11 (PMC4603332; doi:10.1186/1471-2164-16-S10-S11)

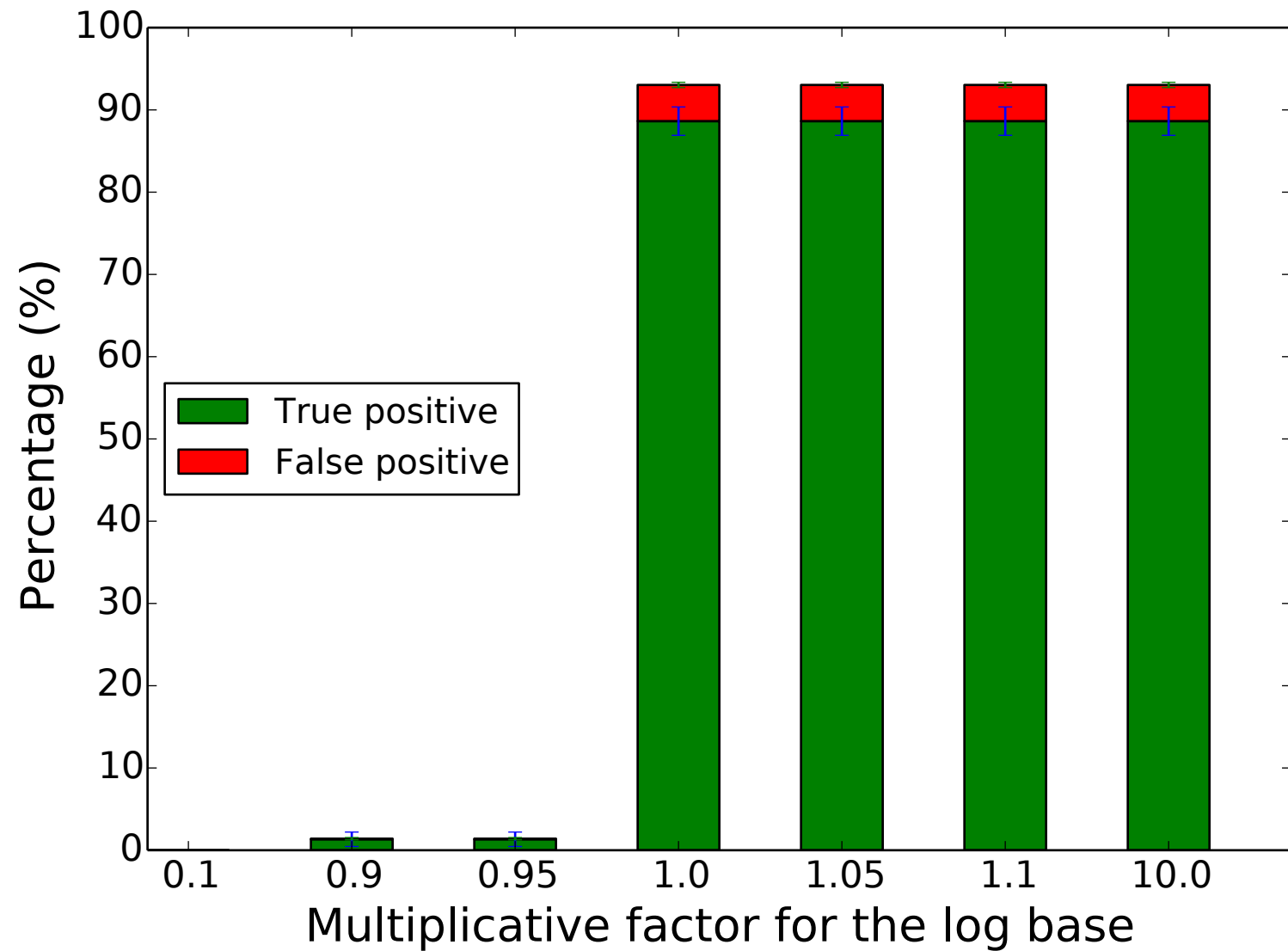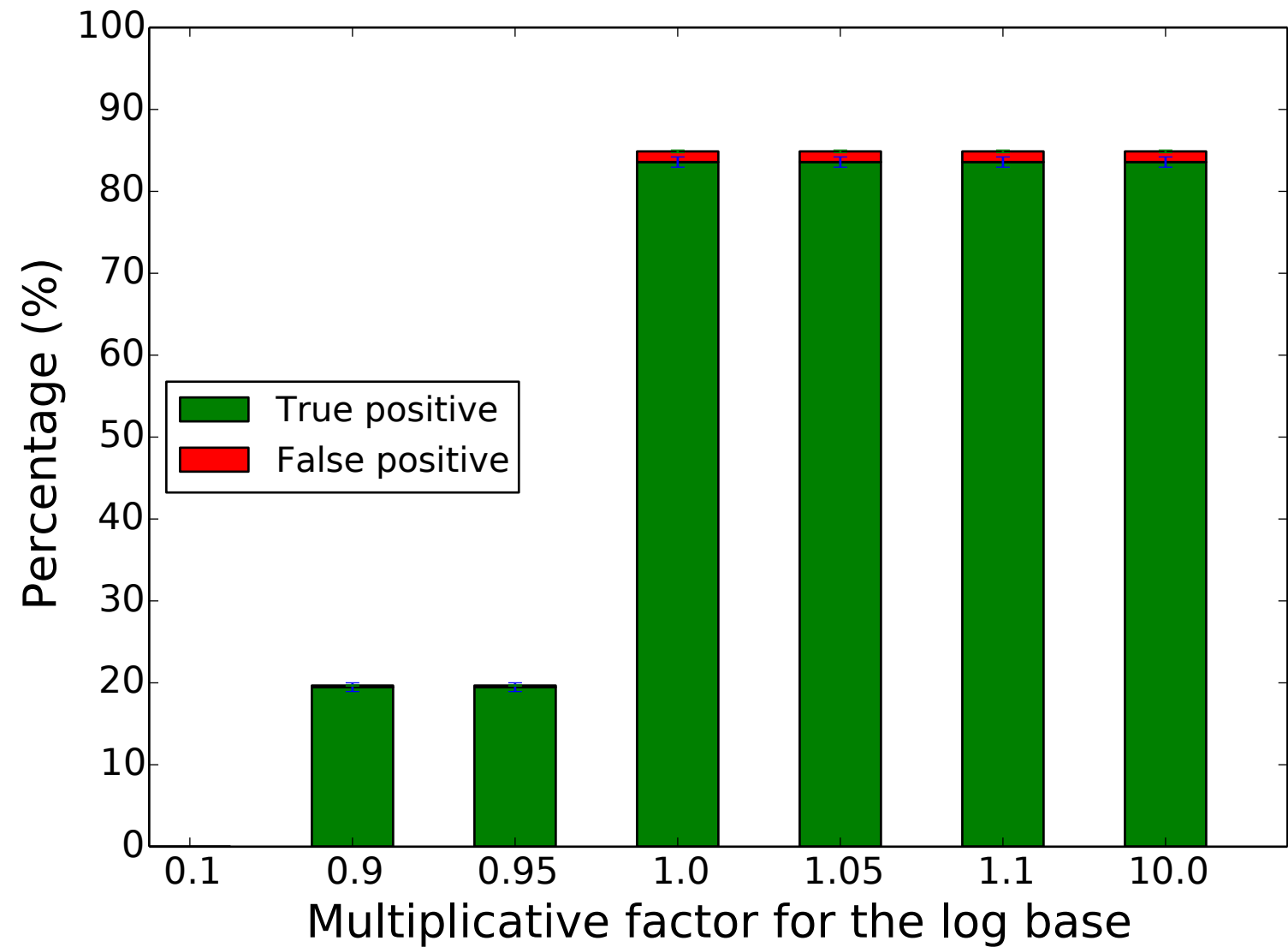

Supplement: Additional File 2 — Figure 11 Effect of number of adjacencies breaks on adjacencies recover in function of base log value. On the left, graph represents the number of adjacencies recovery in function of multiplicative factor for the log base for 50 simulated breaks in each species of 7 tetrapods dataset. On the right, the same graph but with 1050 simulated breaks for each species. As we can see the histogram profile is quietly similar between these two experiments and the one with 550 simulated breaks (see Figure 2). In conclusion, Number of adjacencies breaks didn't impact the optimal value for the log base. (Values are available in Table 1. [file 1471-2164-16-S10-S11-S2.pdf]
